# Reply to Rollin et al.: Clarifying the multifactorial origins of racial disparities in uterine serous carcinoma

**DOI:** 10.1073/pnas.2419163121

**Published:** 2024-10-14

**Authors:** J. J. Kim, M. Simon, G. Fleming

**Affiliations:** ^a^Division of Reproductive Science, Obstetrics and Gynecology, Northwestern University Feinberg School of Medicine, Chicago, IL 60126; ^b^Obstetrics and Gynecology, Northwestern University Feinberg School of Medicine, Chicago, IL 60126; ^c^Division of Gynecologic Oncology, Obstetrics and Gynecology, University of Chicago, Chicago, IL 60637

We thank Rollin et al. for their thoughtful commentary (1) on our manuscript exploring the tumor transcriptomes and immune responses in uterine serous carcinoma (USC) between Black and White patient groups (2). We deeply appreciate your engagement with the research, particularly your emphasis on the importance of acknowledging structural and social factors that may drive observed differences in molecular pathways and gene expression.

We completely agree that race is a sociopolitical construct and not a proxy for genetic variation. Goel et al. (3) published a useful review highlighting the numerous ways in which neighborhood disadvantage interacts with cancer outcomes, including through its influence on biological processes such as gene regulation and epigenetics. Our study (2) demonstrated transcriptomic differences in the tumors from self-reported Black and White patients; however, the causes of these differences remain unclear and are most likely multifactorial, stemming from social determinants of health, structural racism, and molecular factors. In the field of endometrial cancer, investigating these complex interactions is still in its early stages.

Currently, ongoing studies extend our reported findings by incorporating both ancestry and socioenvironmental factors, including structural racism. We aim to carefully contextualize our findings to reflect that observed disparities are likely influenced by a complex interplay of structural inequities and environmental exposures, while also exploring ancestral differences. We are mindful of the need to interpret our data clearly, ensuring that we do not inadvertently suggest that racial disparities are rooted in inherent biological differences.

Ancestry can be determined through an individual’s genetic information (4). Genetic admixture, the result of recent interbreeding between previously separated populations, is typically measured as the overall proportion of a particular ancestry, such as African ancestry, across the genome. African Americans, for instance, have a mix of African and European ancestry, which varies widely, ranging from less than 50% to nearly 100% African ancestry (global ancestry proportion) (5). We posit that, as an additional source of variation, ancestry may influence the phenotype of interest. This aspect of genome biology has not been extensively explored in gynecological cancers, and we anticipate that our ongoing investigations will shed light on the roles of both ancestry and socioenvironmental factors in the USC disparities observed between African American and Non-Hispanic White populations in the United States.

Once again, we thank you for your insightful feedback, which will undoubtedly help strengthen future work in this important area.
